# Supplementary material for: The Pork Meat or the Environment of the Production Facility? The Effect of Individual Technological Steps on the Bacterial Contamination in Cooked Hams
Source: Microorganisms. 2022 May 27;10(6):1106. doi: 10.3390/microorganisms10061106 (PMC9229742; doi:10.3390/microorganisms10061106)
Supplement: Supplementary file 1 [file microorganisms-10-01106-s001.zip › microorganisms-1721849-supplementary.pdf]

Table S1A: Results of determination of the total viable count (TVC), bacteria of the family *Enterobacteriaceae* (ENT) and lactic acid bacteria (LAB) during incubation at 6.5 and 15 °C in samples of meat (M), massaged meat (T), and cooked hams (S). Results are given in log CFU/g.

**Analyzed sample**	**6.5 °C**				**15 °C**			
	**TVC**	**ENT**	**LAB**	**Identified species of LAB**	**TVC**	**ENT**	**LAB**	**Identified species of LAB**
**M1/1**	4,23	<1	<1,699	-	4,204	2	<1,699	-
**M1/2**	2,431	<1	<1,699	-	2,602	<1	<1,699	-
**M1/3**	4,23	1,301	<1,699	-	4,114	1,903	<1,699	-
**M1/4**	3,991	1,176	<1,699	-	3,968	1,699	<1,699	-
**M1/5**	4,146	<1	<1,699	-	4,591	3,342	<1,699	-
**M1/6**	4,079	1,301	<1,699	-	4,613	2,322	<1,699	*Latilactobacillus curvatus*
**M1/7**	2,978	<1	<1,699	-	2,903	<1	<1,699	-
**M1/8**	4,114	<1	<1,699	-	3,663	1,301	<1,699	-
**M1/9**	4,204	1,301	<1,699	-	4,255	2,301	<1,699	-
**M1/10**	4,23	1,477	<1,699	-	4,301	<1	<1,699	-
**T1/1**	4,146	1,602	2,477	species not identified	4,771	3,857	3	*Latilactobacillus curvatus*
**T1/2**	4,477	2,342	3,431	species not identified	4,763	3,919	2,176	*Leuconostoc inhae, Leuconostoc carnosum, Leuconostoc mesenteroides*
**T1/3**	4,255	2,38	2,544	species not identified	4,623	3,875	3	species not identified
**T1/4**	4,491	2,672	<1,699	-	5	3,806	3,114	*Latilactobacillus curvatus*
**T1/5**	4,279	3,23	<1,699	-	4,447	3,732	3,114	*Leuconostoc carnosum, Leuconostoc mesenteroides*
**T1/6**	4,301	2,38	<1,699	-	5	4,602	2,699	*Leuconostoc carnosum*
**T1/7**	4,204	2,756	3,146	species not identified	4,944	4	3,255	*Leuconostoc carnosum, Leuconostoc gelidum*
**T1/8**	4,732	2,255	<1,699	-	4,826	4,041	3,176	*Paucilactobacillus oligofermentans*
**T1/9**	3,778	2,763	<1,699	-	5,041	4,322	2,699	*Leuconostoc carnosum*
**T1/10**	3,716	2,544	<1,699	-	4,763	4,176	3,176	species not identified
**S1/1**	<1	<1	<1,699	-	<1	<1	<1,699	-
**S1/2**	<1	<1	<1,699	-	<1	<1	<1,699	-
**S1/3**	<1	<1	<1,699	-	<1	<1	<1,699	-
**S1/4**	<1	<1	<1,699	-	<1	<1	<1,699	-
**S1/5**	<1	<1	<1,699	-	<1	<1	<1,699	-
**S1/6**	<1	<1	<1,699	-	<1	<1	<1,699	-

Table S1B: Results of determination of the total viable count (TVC), bacteria of the family *Enterobacteriaceae* (ENT), and lactic acid bacteria (LAB) during incubation at 6.5 and 15 °C in samples of meat (M), massaged meat (T), and cooked hams (S). Results are given in log CFU/g.

**Analyzed sample**	**6.5 °C**				**15 °C**			
	**TVC**	**ENT**	**LAB**	**Identified species of LAB**	**TVC**	**ENT**	**LAB**	**Identified species of LAB**
**M2/1**	2,431	1	<1,699	-	2,813	2	<1,699	-
**M2/2**	4,146	2,146	2	species not identified	4,491	<1	2,176	*Latilactobacillus sakei*
**M2/3**	<1	<1	<1,699	-	<1	<1	<1,699	-
**M2/4**	2,447	<1	<1,699	-	2,633	<1	<1,699	-
**M2/5**	2,613	<1	<1,699	-	2,724	<1	<1,699	-
**M2/6**	4,342	2,041	<1,699	-	4,204	3,146	2,519	*Latilactobacillus sakei*
**M2/7**	4,342	1,813	3,079	*Latilactobacillus sakei*	4,255	3,699	2,623	*Latilactobacillus sakei*
**M2/8**	4,146	2,176	1,699	*Latilactobacillus sakei*	3,982	2,987	1,699	*Latilactobacillus sakei*
**M2/9**	4,114	2,431	1,699	*Latilactobacillus sakei*	3,908	2,82	2,519	*Latilactobacillus sakei*
**M2/10**	3,38	1,477	2	species not identified	3,798	2,342	<1,699	_-
**T2/1**	3,531	1,602	2,633	*Leuconostoc carnosum*	3,892	<2	2,886	*Leuconostoc carnosum*
**T2/2**	3,623	<1	<1,699	-	4,23	<2	3,146	*Lentilactobacillus kefiri*<br>*Lentilactobacillus otakiensis*
**T2/3**	4,146	<1	2	species not identified	4,491	<2	3,146	*Leuconostoc carnosum*
**T2/4**	3,944	1,398	2,74	*Leuconostoc gelidum*	4,204	<2	3,114	species not identified
**T2/5**	4,114	<1	3,041	*Leuconostoc carnosum*	3,903	2	3,146	*Leuconostoc carnosum*<br>*Leuconostoc mesenteroides*
**T2/6**	4,146	<1	2,881	*Latilactobacillus sakei*	4,079	<2	3,079	*Leuconostoc carnosum*
**T2/7**	4,079	1,74	3,041	*Leuconostoc carnosum*	4,279	<2	3,114	*Leuconostoc carnosum*
**T2/8**	3,875	<1	2,908	*Leuconostoc carnosum*<br>*Leuconostoc gelidum*	4,23	<2	2,978	*Leuconostoc carnosum*<br>*Latilactobacillus sakei*<br>*Lentilactobacillus kefiri*
**T2/9**	3,613	<1	2,301	-	3,591	<2	2,301	*Leuconostoc carnosum*
**T2/10**	4,176	<1	2,602	*Leuconostoc gelidum*	4,362	<2	3,176	*Leuconostoc carnosum*<br>*Leuconostoc mesenteroides*<br>*Latilactobacillus sakei*
**S2/1**	<1	<1	<1,699	-	<1	<1	<1,699	-
**S2/2**	<1	<1	<1,699	-	<1	<1	<1,699	-
**S2/3**	<1	<1	<1,699	-	<1	<1	<1,699	-
**S2/4**	<1	<1	<1,699	-	<1	<1	<1,699	-
**S2/5**	1,398	<1	<1,699	-	1,477	<1	<1,699	-
**S2/6**	<1	<1	<1,699	-	<1	<1	<1,699	-

Table S1C: Results of determination of the total viable count (TVC), bacteria of the family *Enterobacteriaceae* (ENT), and lactic acid bacteria (LAB) during incubation at 6.5 and 15 °C in samples of meat (M), massaged meat (T), and cooked hams (S). Results are given in log CFU/g.

**Analyzed sample**	**6.5 °C**				**15 °C**			
	**TVC**	**ENT**	**LAB**	**Identified species of LAB**	**TVC**	**ENT**	**LAB**	**Identified species of LAB**
**M3/1**	3,255	<1	<1,699	-	3,279	<1	<1,699	
**M3/2**	4,708	1,74	<1,699	-	4,491	1,929	2,477	*Leuconostoc gelidum*<br>*Enterococcus gilvus*
**M3/3**	3,806	<1	<1,699	-	3,653	1,699	1,699	*Enterococcus gilvus*
**M3/4**	3,146	<1	<1,699	-	3,041	<1	<1,699	-
**M3/5**	4,041	1,301	<1,699	-	3,908	1,813	<1,699	-
**M3/6**	4,146	<1	<1,699	-	3,934	1,845	<1,699	-
**M3/7**	3,681	<1	<1,699	-	3,38	1,544	<1,699	-
**M3/8**	3,748	<1	<1,699	-	3,771	1,788	<1,699	-
**M3/9**	3,748	1,301	<1,699	-	3,94	1,544	<1,699	-
**M3/10**	3,477	<1	<1,699	-	3,491	<1	<1,699	-
**T3/1**	3,519	1,477	2,74	*Leuconostoc carnosum*<br>*Leuconostoc gelidum*<br>*Latilactobacillus fuchuensis*	3,892	<2	2,826	species not identified
**T3/2**	3,792	1,903	2,74	species not identified	4,176	<2	2,886	*Leuconostoc carnosum*
**T3/3**	3,699	1,699	2,74	*Leuconostoc carnosum*<br>*Leuconostoc gelidum*	4,255	<2	3,079	*Leuconostoc gelidum*
**T3/4**	3,653	1,653	2,851	*Leuconostoc gelidum*<br>*Leuconostoc inhae*	3,978	<2	2,869	*Leuconostoc gelidum*
**T3/5**	3,886	1,954	2,398	*Latilactobacillus sakei*	4,255	2	3,079	*Leuconostoc inhae*
**T3/6**	3,908	2,079	2,653	species not identified	3,886	<2	3,114	*Leuconostoc carnosum*
**T3/7**	3,663	1,875	3,146	*Leuconostoc carnosum*<br>*Leuconostoc gelidum*	4,114	<2	3,114	*Leuconostoc mesenteroides*<br>*Latilactobacillus curvatus*
**T3/8**	3,908	1,653	2,813	*Leuconostoc gelidum*<br>*Latilactobacillus curvatus*	4,176	2,301	3,204	*Latilactobacillus curvatus*
**T3/9**	4	1,544	2,544	species not identified	4,301	<2	3,23	*Leuconostoc carnosum*
**T3/10**	4	1,845	2,699	species not identified	4,301	<2	3,301	*Leuconostoc carnosum*<br>*Leuconostoc gelidum*<br>*Lactococcus lactis*
**S3/1**	<1	<1	<1,699	-	<1	<1	<1,699	-
**S3/2**	<1	<1	<1,699	-	<1	<1	<1,699	-
**S3/3**	<1	<1	<1,699	-	<1	<1	<1,699	-
**S3/4**	2,987	<1	<1,699	-	2,908	<1	<1,699	-
**S3/5**	<1	<1	<1,699	-	<1	<1	<1,699	-
**S3/6**	<1	<1	<1,699	--	<1	<1	<1,699	-

Table S1D: Results of determination of the total viable count (TVC), bacteria of the family *Enterobacteriaceae* (ENT), and lactic acid bacteria (LAB) during incubation at 6.5 and 15 °C in samples of meat (M), massaged meat (T), and cooked hams (S). Results are given in log CFU/g.

**Anayzed sample**		**6.5 °C**				**15 °C**		
	**TVC**	**ENT**	**LAB**	**Identified species of LAB**	**TVC**	**ENT**	**LAB**	**Identified species of LAB**
**M4/1**	3,447	1,301	<1,699	-	3,82	1,653	<1,699	-
**M4/2**	2,929	<1	<1,699	-	3,079	<1	<1,699	-
**M4/3**	3,785	<1	<1,699	-	3,934	1,398	<1,699	-
**M4/4**	3,114	<1	<1,699	-	3,724	<1	<1,699	-
**M4/5**	3,38	<1	<1,699	-	3,568	<1	<1,699	-
**M4/6**	3,23	<1	<1,699	-	3,146	1,301	<1,699	-
**M4/7**	3,903	<1	<1,699	-	3,826	<1	<1,699	-
**M4/8**	3,114	<1	<1,699	-	3,255	<1	<1,699	-
**M4/9**	3,114	<1	<1,699	-	3,204	<1	<1,699	-
**M4/10**	3,771	<1	<1,699	-	3,82	1,301	<1,699	-
**T4/1**	4,079	<1	3,763	*Leuconostoc carnosum*<br>*Dellaglioa algida*	4	2,176	3,748	species not identified
**T4/2**	4,041	<1	3,653	*Leuconostoc gelidum*<br>*Dellaglioa algida*	4,146	<2	3,699	*Latilactobacillus fuchuensis*
**T4/3**	4,146	<1	3,681	*Latilactobacillus curvatus*	4,477	<2	3,724	*Leuconostoc mesenteroides*
**T4/4**	4,041	<1	3,756	*Latilactobacillus curvatus*<br>*Dellaglioa algida*	4,114	2,398	3,944	*Leuconostoc mesenteroides*<br>*Leuconostoc gelidum*
**T4/5**	4,204	<1	3,845	*Leuconostoc gelidum*<br>*Dellaglioa algida*	4,491	<2	3,857	*Leuconostoc carnosum*
**T4/6**	4,079	<1	3,833	*Leuconostoc gelidum*<br>*Dellaglioa algida*	4,477	<2	3,792	*Latilactobacillus sakei*
**T4/7**	4,176	<1	3,716	*Latilactobacillus sakei*	4,146	<2	3,74	*Leuconostoc gelidum*
**T4/8**	4,079	<1	3,643	species not identified	4,176	2,301	3,699	*Leuconostoc gelidum*<br>*Latilactobacillus sakei*
**T4/9**	4,342	<1	3,58	*Dellaglioa algida*	4,491	2,477	3,699	*Leuconostoc mesenteroides*
**T4/10**	4,255	<1	3,806	*Latilactobacillus sakei*	4,633	<2	3,792	*Leuconostoc mesenteroides*
**S4/1**	<1	<1	<1,699	-	<1	<1	<1,699	-
**S4/2**	<1	<1	<1,699	-	<1	<1	<1,699	-
**S4/3**	<1	<1	<1,699	-	<1	<1	<1,699	-
**S4/4**	<1	<1	<1,699	-	<1	<1	<1,699	-
**S4/5**	<1	<1	<1,699	-	<1	<1	<1,699	-
**S4/6**	<1	<1	<1,699	-	<1	<1	<1,699	-

Table S1E: Results of determination of the total viable count (TVC), bacteria of the family *Enterobacteriaceae* (ENT), and lactic acid bacteria (LAB) during incubation at 6.5 and 15 °C in samples of meat (M), massaged meat (T), and cooked hams (S). Results are given in log CFU/g.

**Analyzed sample**	**6.5 °C**				**15 °C**			
	**TVC**	**ENT**	**LAB**	**Identified species of LAB**	**TVC**	**ENT**	**LAB**	**Identified species of LAB**
**M5/1**	2,602	<1	<1,699	-	3,204	1,176	<1,699	-
**M5/2**	1,813	<1	<1,699	-	2,732	1	<1,699	-
**M5/3**	<1	<1	<1,699	-	<1	<1	<1,699	-
**M5/4**	2,699	<1	<1,699	-	3,079	1,477	<1,699	-
**M5/5**	1,398	<1	<1,699	-	2,431	<1	<1,699	-
**M5/6**	<1	<1	<1,699	*Leuconostoc carnosum*	2,898	<1	<1,699	-
**M5/7**	1,954	<1	<1,699	-	2,778	<1	<1,699	-
**M5/8**	2,653	<1	2,301	*Leuconostoc carnosum*	3,301	<1	<1,699	-
**M5/9**	1,301	<1	<1,699	-	3,041	<1	<1,699	-
**M5/10**	2,23	<1	1,699	species not identified	2,94	<1	<1,699	-
**T5/1**	4,176	0,699	2,653	*Leuconostoc carnosum*<br>*Leuconostoc gelidum*<br>*Latilactobacillus fuchuensis*	4	2,74	2,58	*Leuconostoc carnosum*<br>*Leuconostoc gelidum*<br>*Latilactobacillus sakei*
**T5/2**	4,415	1,74	2,881	*Leuconostoc gelidum*<br>*Latilactobacillus sakei*	4,362	2,929	2,934	*Leuconostoc gelidum*<br>*Latilactobacillus sakei*
**T5/3**	4,568	<1	2,991	*Leuconostoc gelidum*<br>*Latilactobacillus sakei*	4,362	2,954	3,114	*Leuconostoc carnosum*<br>*Leuconostoc mesenteroides*<br>*Latilactobacillus sakei*
**T5/4**	4,477	<1	3,114	*Leuconostoc gelidum*	4,362	3,146	3,041	*Leuconostoc carnosum*
**T5/5**	4,491	<1	3,146	*Leuconostoc gelidum*<br>*Latilactobacillus fuchuensis*	4,342	2,978	3,279	*Leuconostoc carnosum*<br>*Latilactobacillus sakei*
**T5/6**	4,146	<1	2,881	*Leuconostoc carnosum*<br>*Leuconostoc gelidum*<br>*Latilactobacillus sakei*	4,322	2,929	3,114	*Leuconostoc carnosum*<br>*Leuconostoc inhae*<br>*Latilactobacillus sakei*
**T5/7**	4,591	<1	3	*Leuconostoc gelidum*<br>*Latilactobacillus sakei*	4,146	2,845	3,279	*Leuconostoc carnosum*<br>*Latilactobacillus sakei*
**T5/8**	4,544	<1	3,114	*Leuconostoc gelidum*<br>*Latilactobacillus sakei*	4,38	2,398	3	*Latilactobacillus sakei*
**T5/9**	4,544	<1	3	*Latilactobacillus sakei*	4,342	2,845	3,114	*Leuconostoc gelidum*<br>*Latilactobacillus sakei*
**T5/10**	4,531	<1	3	*Leuconostoc gelidum*<br>*Latilactobacillus sakei*	4,301	2,079	3,114	*Leuconostoc carnosum*
**S5/1**	<1	<1	<1,699	-	<1	<1	<1,699	-
**S5/2**	<1	<1	<1,699	-	<1	<1	<1,699	-
**S5/3**	<1	<1	<1,699	-	<1	<1	<1,699	-
**S5/4**	<1	<1	<1,699	-	<1	<1	<1,699	-

**S5/5**	<1	<1	<1,699	-	<1	<1	<1,699	-
**S5/6**	<1	<1	<1,699	-	<1	<1	<1,699	-

Table S2: Results of determination of the total viable count (TVC), bacteria of the family *Enterobacteriaceae* (ENT), and lactic acid bacteria (LAB) during incubation at 15 °C/7 d in samples of cooked hams after a stability test (ST). Results are given in log CFU/g.

Analyzed sample	TVC	ENT	LAB	Identified species of LAB
**ST1**	3,519	1,845	2,959	*Leuconostoc mesenteroides*
**ST2**	4,964	3	4,041	*Leuconostoc mesenteroides*<br>*Latilactobacillus curvatus*<br>*Lactococcus lactis*
**ST3**	<2	<1	<1,699	-
**ST4**	4,643	3,079	4,114	*Leuconostoc mesenteroides*<br>*Leuconostoc carnosum*
**ST5**	3,763	2,462	3,431	*Lactococcus lactis,*<br>*Latilactobacillus sakei*
**ST6**	4,279	2,839	3,991	-
**ST7**	3,519	<1	3,041	-
**ST8**	4,74	2,447	4,763	*Leuconostoc gelidum*
**ST9**	2,892	<1	2,176	*Leuconostoc carnosum*
**ST10**	4,477	<1	3,845	*Leuconostoc carnosum,*<br>*Lactococcus lactis*
**ST11**	2,716	<1	2,176	-
**ST12**	4,146	<1	3,602	*Leuconostoc carnosum,*<br>*Lactococcus lactis*
